# Effects of linear periodization of combined training on quality of life of adults with obesity: a blind randomized controlled trial

**DOI:** 10.1038/s41598-022-06461-8

**Published:** 2022-02-16

**Authors:** Willen Remon Tozetto, Larissa dos Santos Leonel, Tiago Turnes, Giovani Firpo Del Duca

**Affiliations:** 1 Sports Center, Federal University of Santa Catarina, Florianópolis, Santa Catarina Brazil; 2 Centro de Desportos, Núcleo de Pesquisa em Exercício Físico e Doenças Crônicas Não Transmissíveis, Campus Universitário Reitor João David Ferreira Lima, Florianopolis, SC CEP: 88040-900 Brazil

**Keywords:** Obesity, Quality of life

## Abstract

This study aimed to compare the effect of 16-weeks of combining aerobic and strength training with a linear increase or fixed intensity on the health-related quality of life (HRQoL) of obese adults. This single-blinded clinical trial involved adults with obesity (BMI ≥ 30 kg/m^2^), randomized into control (CG), fixed intensity (FG), or linear increase (LG) groups. The FG and LG performed 16 weeks of combined (aerobic + strength) training for 60 min, three times a week. The FG performed aerobic exercises between 50 and 59% of the heart rate reserve (HRres) and strength at 10–12 maximum repetitions (RM). The LG started with 40–49% of HRres and 12–14 RM and progressively increased the intensity (50–59% and 10–12 RM; 60–69% and 8–10 RM). The HRQoL was assessed using the SF-36 questionnaire. Generalized estimation equations and mean differences (∆) were used. Of the 69 participants (23 per group), 36 completed the intervention (CG = 13, FG = 9, and LG = 14). A significant difference was observed in the time of the physical function, with superiority in the training groups (CG: ∆ = 1.2 vs. FG and LG, respectively: ∆ = 10.0). The mental health component and mental health domain showed significant differences for the FG (∆ = 30.2 and ∆ = 23.1, respectively). In conclusion, the combined training improved physical functioning. Specifically, fixed-intensity training effectively enhanced mental health indicators.

**Trial Registration:** This study is registered at www.ensaiosclinicos.gov.br/ (No. RBR-3c7rt3), Date of registration: 07/02/2018.

## Introduction

Obesity is one of the main chronic non-communicable diseases (CNCD) in the modern world, caused by a positive energy balance and associated with multiple factors such as inadequate eating habits, low levels of physical activity, and a stressful lifestyle^1^. This global epidemic affects 13% of the world population^1^ and 22.6% of the Brazilian population, placing Brazil as one of the ten countries with the highest rate of the disease^2^. Obesity increases family expenses by 15% to 195% for medications, consultations, tests, hospitalizations, and treatment in Brazil^3^. Data from the Brazilian Institute of Geography and Statistics (IBGE) show that in public health, more than 487 million reais are spent annually on hospitalizations and outpatient procedures directly related to obesity in the Brazilian population^4^. Among the health consequences are the increased risk of various cardiovascular, endocrine, respiratory, and musculoskeletal disorders, in addition to serious diseases such as type II diabetes mellitus, arterial hypertension and non-alcoholic fatty liver^1,5,6^. The increase prevalence of obesity has direct consequences on the health-related quality of life (HRQoL)^7^, together with increased morbidity and mortality^8^.

Excess body fat is closely linked to physical and psychosocial losses, with a dose–response effect on the HRQoL^9,10^. Obesity can cause functional limitations, reduced work capacity, and subsequent psychological problems with increased anxiety, obsessive–compulsive disorder, paranoid personality disorder, and depression^11^. To mitigate the damage caused by obesity and improve the HRQoL, several strategies are used (e.g., nutritional, psychological, drug, surgical treatment^12^). In this sense, numerous studies have been conducted in this population, evaluating from the consumption of milk and eggs^13,14^, to medicinal fruits and their implications in weight reduction and improving insulin resistance^15^. However, regular physical exercise and training among the most effective for improving various health parameters in this population^8,16^.

Among the numerous methods available, combined training, namely, combining aerobic and muscle strength exercises, is more effective for attenuating the losses from obesity when compared to aerobic or strength training performed in isolation^17^, and with greater benefits in physical and mental health^8,16,18^, which could contribute to an improved HRQoL This was recently corroborated by the European Association for the Study of Obesity (EASO), who recommended with a “B” level of evidence, combined training for improving insulin sensitivity, cardiorespiratory parameters and muscle strength from an “A” level of evidence, and improving the physical component of the HRQoL of those with obesity^19^.

As noted in isolated aerobic or strength training, exercise progression seems to be an important consideration relating to the benefits. Hence, the increase in the intensity of isolated exercise is superior in improving health parameters related to obesity, compared to training without progression. For example, the improvement of metabolic profile, reduction of chronic pain, release of monoamines such as serotonin, associated with good mood, distracting negative thoughts, and consequently adherence to training, contributing to physical and psychological well-being^20–23^. However, lower intensities provided greater gains in improving HRQoL^24^, although not unanimously^25^. In addition, a direct longitudinal study comparing regimens with a progressive linear increase in exercise intensity versus a fixed intensity during combined training is still lacking. The effects of exercise intensity progression during combined training on HRQOL remains unknown. It is important to note that a gradual increase in intensity was recommended by the EASO^19^. Nevertheless, EASO makes this recommendation according to an analogy with the general population, as there is a scarcity of specific studies that focused on the distinct nature of this population. Furthermore, studies comparing combined training with aerobic and strength training alone and not with different intensity manipulations^26–28^. Thus, the effects of exercise intensity progression during combined training are still scarce in relation to the HRQoL of populations with CNCDs, such as obesity.

Therefore, this study aimed to compare the effects of 16 weeks of combined training with fixed intensity or linearly increasing intensity on the health-related quality of life of obese adults. Considering that combined training enhances parameters related to the health of those with obesity^20–22^ and moderate or high intensities may initially cause greater discomfort, such as joint pain and excessive tiredness^21^ for beginners, it was hypothesized that the increase in structured intensity during combined training may enhance the HRQoL of individuals with obesity^26–28^ compared with a fixed intensity. Thus, initially adopting lower intensities could indirectly improve the participants’ perception of HRQoL in this study.

## Methods

### Study design

A single-blind randomized controlled trial with three groups of obese individuals was conducted. Participants performed a series of assessments and answered the SF-36 questionnaire for HRQoL analysis before and after a 16-week intervention period, in which they were divided into three groups: control group (CG), fixed intensity group (FG), and linearly increasing intensity group (LG). For this purpose, volunteers residing in the metropolitan area of Florianópolis, Santa Catarina State, Brazil were recruited. Methodological details can be found in the study protocol article^29^. This study was approved by the Human Research Ethics Committee of the Federal University of Santa Catarina (2.448.674) and registered in the Brazilian Registry of Clinical Trials (RBR-3c7rt3). All participants were duly informed about the procedures and provided signed informed consent. The entire process was carried out in accordance with the ethical principles of the Declaration of Helsinki.

### Participants

We selected men and women aged 20 to 50 years with a body mass index (BMI) between 30 and 39.9 kg/m^2^ (degree obesity I and II). Inclusion criteria were adults who did not exercise weekly more than twice in the past 3 months, non-smokers, did not consume excessive alcohol (≥ 7 drinks and ≥ 14 weekly drinks for women and men, respectively)^30^, no osteoarticular pathology limiting the practice of physical exercises, not on medication to control and/or treat obesity, no history of weight-loss surgical procedures, were eligible for this study, and had other diseases besides obesity. Participants who formally withdrew from the study, who did not complete the questionnaires, or who changed the habits observed in the eligibility criteria at the end of the study, were excluded.

After signing the informed consent, all participants underwent a series of evaluations. The allocation was stratified by sex, age, and BMI, collected at baseline, with a ratio of 1:1:1 through an online platform (www.randomized.org). Independent researchers who were not involved in the evaluations and interventions conducted this process. The allocation list was unveiled to the coaches only on the start date of the intervention. All study procedures were conducted between March and November 2018. Due to the impossibility of blinding the participants and professionals who conducted the training, this study was able to blind only the evaluations, which were carried out by professionals who did not participate in the training, without distinction between the groups.

### Interventions

LG and FG participated in 16 weeks of combined training (aerobic and muscle strength in the same session). Aerobic training was performed continuously by walking and/or running on the athletic track, with intensities prescribed based on the reserve heart rate (HRres). For the training groups, aerobic exercise was performed continuously by walking or running on a synthetic 400-m running track. Exercise intensity was monitored individually using a portable HR chest belt (POLAR, S810i, Finland). HRres readjustment was performed at each of the 5-week mesocycles for all groups. Strength training was performed in multiple sets, using six exercises involving large muscle groups in the following order: barbell bench press, seated pec deck fly, low row, pull-down, barbell squat, and leg press 45°, with prescription for maximum repetition ranges (RM). The participants were instructed to perform the exercises at a maximum amplitude of range of motion at a self-selected pace. The established weekly frequency was three non-consecutive times, and no criteria were established for the exclusion of participants in case of low adherence. The training lasted an average of 60 min, with the first half being dedicated to aerobic training. The participants were divided into two forms of periodization. All sessions started with 5 min of aerobic warm-up and ended with 5 min of stretching or muscle relaxation.

The LG participated in a linearly increasing intensity training, divided into three mesocycles of 5 weeks each, progressing between light intensities (40–49% HRres/12–14 RM), moderate (50–59% HRres/10–12 RM), and vigorous (60–69% HRres/8–10 RM). In the FG, the intensity remained moderate (50–59% HRres/10–12 RM) throughout the study. The first week was used for training familiarization for both groups (30–39% HRres/10–15 RM). All participants in the training groups were instructed to maintain their daily physical activities (i.e., displacement) or their diet during the intervention, modifying only the training over 16 weeks. The CG did not receive any intervention and was instructed to maintain their daily living activities and not change their habits.

### Assessment for sample characterization and exercise prescription

Before and after the intervention, the participants completed an online questionnaire and provided sociodemographic information, including sex (male and female), marital status (with and without partner), ethnicity (white or others), education (in years of study), and age (in complete years). Body composition was assessed using the tetrapolar electrical bioimpedance (IN BODY 720, OTTOBONI, Rio de Janeiro, Brazil), handled by experienced evaluators following standard guidelines^31^. To prescribe aerobic training by HRres, the maximum and resting heart rates were used to calculate the ideal training zone, which was obtained using portable HR meters (POLAR, S810i). The maximum heart rate was derived using the incremental test described by Libardi et al.^18^. The resting HR was measured while the participant was lying down with the frequency meter strap positioned. Three one-minute notes were made with a one-minute interval between them. The reference value is the average of the measurements observed at different time points. Resting HR reassessments were performed at the end of each mesocycle to adjust the intensity.

### Outcome assessment

The HRQoL was measured using the SF-36 questionnaire developed by Ware and Sherbourne. The version presented to the participants was translated and validated in Portuguese by Ciconelli et al.^32^. Each participant completed the questionnaire through the online platform, Question Pro^®^, pre-intervention and at the end of 16 weeks. This questionnaire evaluates the HRQoL through 36 questions involving eight domains to separately evaluate each aspect of the construct. These domains are analyzed according to the physical and mental component summary, but the individual use of the domains is emphasized to better understand the responses. The Physical Component Summary (PCS) is divided into role-physical, physical functioning, bodily pain, and general health status. On the other hand, the mental component summary (MCS) is separated into role-emotional, social functioning, mental health, and vitality. Its scale ranges from 0 to 100, with higher values representing better HRQoL within each domain or component.

### Statistical analysis

Sample calculation was performed using GPOWER 3.1.7 software, adopting a significance level of 0.05, a power of 80%, and an effect size of 0.18 in repeated measures analysis, with a ratio of 1:1:1 among the three study groups for the main variable of the main project, which is VO_2max_^29^. The calculation yielded a minimum of 26 participants in each group, totaling at least 78 participants.

Sociodemographic variables were used to characterize the sample. Continuous variables were expressed as mean and standard error, and categorical variables as relative frequency. Baseline differences between groups were tested using analysis of variance for independent samples (one-way ANOVA) and chi-square (χ^2^). Data distribution was verified using the Shapiro–Wilk test.

The participants’ HRQol was analyzed who stayed until the end of the study and had all the evaluation data. Intra-and intergroup analyses were performed using generalized estimation equations (GEE) with posthoc Bonferroni correction. Additional analyses are presented in the Supplementary Material. Data are expressed as the mean and standard error, with α = 0.05. Effect size analyses were performed using partial eta-squared (${\upeta }_{\mathrm{p}}^{2}$), considering the interpretation as small (≤ 0.13), medium (0.14 ≥ ${\upeta }_{\mathrm{p}}^{2}$  ≤ 0.25), and large (≥ 0.26)^33^. The magnitude of the (post–pre) difference between the evaluations was expressed as the mean difference (Δ). All analyses were performed using IBM SPSS (version 21.0; IBM CORP., Armonk, NY, USA). GRAPHPAD PRISM 7 was used to illustrate the scores of each participant’s physical and mental components in the pre-and post-intervention moments.

### Ethics approval and consent to participate

The study was approved by the Human Research Ethics Committee of the Federal University of Santa Catarina (2.448.674). All participants were duly informed about the procedures and provided signed informed consent.

## Results

**Figure 1:**
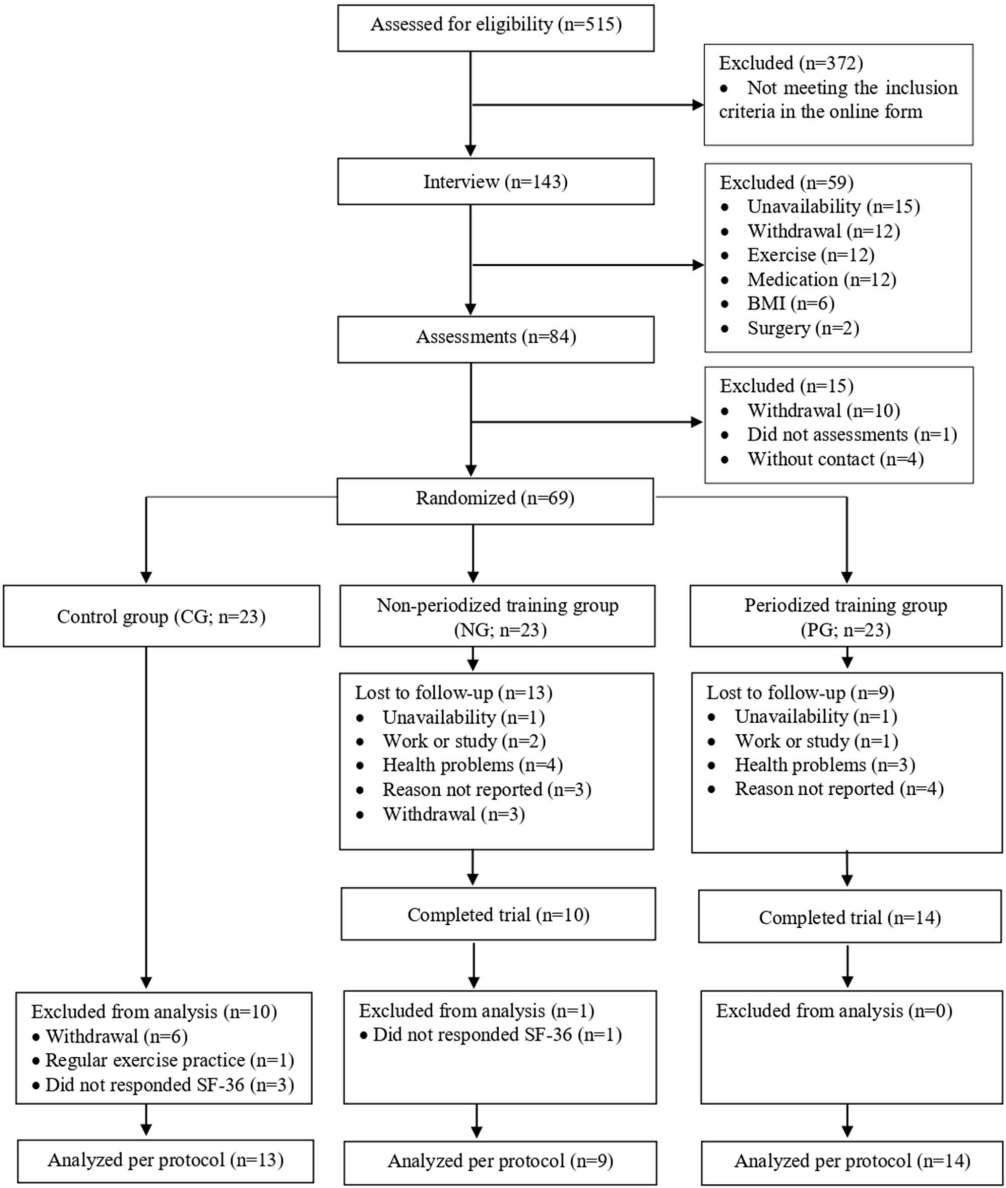
Flowchart of the study process.

The study recruited 515 volunteers. After considering the eligibility criteria, 69 patients were randomized into three groups (control group [CG]: 23; fixed intensity group [FG]: 23; linearly increasing intensity group [LG]: 23). A total of 36 completed all phases of the trial (CG = 13, FG = 9, and LG = 14) and were included in the analyses. With this sample, for the PCS and MCS variables, the effect size of Cohen's *f* was 0.24 and 0.44, offering a sampling power of 70% and 99%, respectively. Figure 1 shows details of this information.

Table 1 presents a comparison of the sociodemographic characteristics of the obese adults whose data were analyzed. No statistically significant differences were found between groups according to the analyzed variables.

**Table 1:** Characteristics of participant who completed the trial (n = 36).

Variable	Control group (n = 13)	Fixed intensity group (n = 9)	Linearly increasing group (n = 14)	p value
	n (%)	n (%)	n (%)	
Sex (female)	8 (36.4%)	6 (27.3%)	8 (36.4%)	0.900
Marital status (with partner)	10 (38.5%)	7 (26.9%)	9 (34.6%)	0.697
Ethnicity (white)	11 (37.9%)	7 (24.1%)	11 (37.9%)	0.897

	$\overline{X }$ (± SE)	$\overline{X }$ (± SE)	$\overline{X }$ (± SE)	
Age (years)	35.2 ± 2.1	37.4 ± 1.3	38.4 ± 1.6	0.388
Education (years)	15.5 ± 0.5	15.2 ± 0.9	16.6 ± 0.8	0.388
BMI (kg/m^2^)	33.1 ± 0.8	32.3 ± 0.8	33.2 ± 0.5	0.739

*n* absolute frequency, *%* relative frequency, $\overline{x }$ = mean, *SE* standard error, *BMI* body mass index.

Participants in the FG and LG groups reached a 67.1% and 58.6% session frequency, respectively, with no difference between the groups (p = 0.343). The frequency of the first mesocycle was 76.3% and 69.5% (p = 0.398), in the second 63.7% and 44.8% (p = 0.113), and in the third, 59.3% and 52.4% (p = 0.476), for the FG and LG, respectively. The prescribed intensity was met throughout the aerobic training sessions by 90% of the participants, with no differences in the total volume of aerobic training (p = 0.657) and strength (p > 0.999) between the groups.

Table 2 presents the HRQoL components and domains. In the PCS and its domains, a significant increase was observed with medium effect size over time only in physical functioning, with an improvement of 10.0 points for the FG and LG, while CG increased only 1.2 points. By analyzing the differences in the values of role-physical and bodily pain, the training groups improved their scores, while the CG worsened. Although somewhat expected, the training group, regardless of periodization, improved in the PCS when compared to the CG (except for general health for the LG). Significant interactions were found in MCS and mental health, with improved scores for the FG. Significant results over time were observed in all MCS domains, with medium to large effect sizes, with the three groups showing an increase in most MCS domains (except for mental health for LG).

**Table 2:** Comparison of the effects of training on Health-related quality of life (n = 36).

SF-36	Pre	Post	Δ	${\upeta }_{\mathrm{p}}^{2}$			p value		
	$\overline{X }$± SE			G	T	G × T	G	T	G × T
**Physical component summary**									
CG	64.2 ± 4.5	61.3 ± 5.3	− 2.9						
FG	59.1 ± 4.9	66.4 ± 3.9	7.3	0.05	0.03	0.06	0.339	0.305	0.328
LG	66.6 ± 4.3	71.5 ± 4.2	4.9						
**Role-physical**									
CG	76.9 ± 6.9	63.5 ± 10.0	− 13.4						
FG	50.0 ± 11.8	55.6 ± 15.1	5.6	0.13	0.01	0.04	0.112	0.975	0.454
LG	75.0 ± 9.8	82.1 ± 8.5	7.1						
**Physical functioning**									
CG	74.6 ± 5.6	75.8 ± 6.2	1.2						
FG	79.4 ± 4.9	89.4 ± 3.5	10.0	0.05	0.17	0.07	0.319	0.007	0.212
LG	74.6 ± 4.6	84.6 ± 3.3	10.0						
**Bodily pain**									
CG	61.5 ± 5.9	61.2 ± 5.9	− 0.3						
FG	61.9 ± 6.7	66.1 ± 6.1	4.2	0.04	0.02	0.01	0.475	0.424	0.749
LG	65.9 ± 5.2	72.1 ± 5.4	6.2						
**General health**									
CG	43.7 ± 6.2	45.0 ± 3.9	1.3						
FG	45.1 ± 5.2	54.4 ± 3.2	9.3	0.03	0.01	0.07	0.621	0.439	0.163
LG	50.9 ± 4.2	47.1 ± 4.4	− 3.8						
**Mental component summary**									
CG	48.0 ± 7.2	59.2 ± 5.4	11.2						
FG	42.3 ± 6.0	72.5 ± 4.0*	30.2	0.03	0.36	0.16	0.678	< 0.001	0.016
LG	56.7 ± 5.9	63.9 ± 6.1	7.2						
**Role-emotional**									
CG	46.2 ± 10.6	64.1 ± 10.5	17.9						
FG	25.9 ± 11.4	74.1 ± 12.6	48.2	0.01	0.25	0.08	0.790	< 0.001	0.167
LG	52.4 ± 11.0	66.7 ± 12.1	14.3						
**Social functioning**									
CG	53.8 ± 7.1	68.3 ± 6.3	14.5						
FG	56.9 ± 7.9	84.7 ± 5.5	27.8	0.04	0.24	0.10	0.387	< 0.001	0.147
LG	64.3 ± 5.8	68.8 ± 6.2	4.5						
**Mental health**									
CG	53.9 ± 7.7	60.0 ± 4.9	6.1						
FG	52.0 ± 6.5	75.1 ± 3.8^#^*	23.1	0.03	0.23	0.25	0.631	< 0.001	< 0.001
LG	64.3 ± 5.6	62.9 ± 6.1	− 1.4						
**Vitality**									
CG	38.1 ± 6.3	44.2 ± 5.4	6.1						
FG	34.4 ± 5.2	56.1 ± 4.1	21.7	0.07	0.32	0.09	0.305	< 0.001	0.164
LG	45.7 ± 4.4	57.1 ± 5.7	11.4						

$\overline{X }$ mean, *SE* standard error, *Δ* difference between post and pre-intervention, ${\eta }_{p}^{2}$ partial eta-squared; *Group (G)* difference between groups, *Time (T*) difference between times, *Group* × *Time (G* × *T)* interaction between time and group, *PCS* physical component summary, *MCS* mental component summary.

*Significant difference pre vs post intra-group; ^#^significant difference with the control group (p ≤ 0.05).

**Figure 2:**
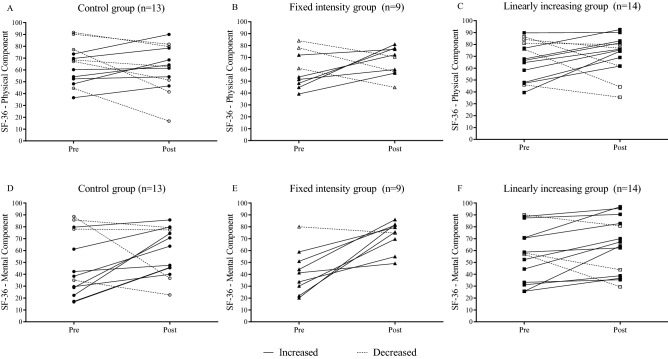
Individual scores in the components of health-related quality of life before and after the intervention period in adults with obesity.

The individual changes before and after the intervention are shown in Fig. 2. In the PCS (A) and MCS (D) of the CG, six (43.9%) and four (69.3%) participants, respectively, showed improvement in scores. In graph B, six participants (66.6%) showed an increase in the PCS score, with the initial score of these individuals being lower than the others. In graph E, the individual with the highest initial score was the only one that reduced his score among the nine participants. In the LG (C; F) representations, nine (64.3%) people improved their PCS scores, while 11 (78.6%) increased MCS scores.

## Discussion

The main objective of this randomized controlled trial was to compare the effects of 16 weeks of fixed intensity and linearly increasing intensity of combined training on the HRQoL in adults with obesity. Improvements were found in the MCS and mental health domain only after fixed intensity training. In addition, greater magnitudes of improvement were found in the MCS domains for the FG compared to the LG and CG, which is pertinent to the improvement of the psychological and social aspects. However, this was not statistically significant. Furthermore, a significant difference in time was observed for the functional capacity domain, with an increase in the score of the groups that underwent training, regardless of periodization.

Obesity is a problem in most developed and developing countries, generating monetary, physical, and mental costs for those involved^1–4,34,35^. For the most part, it has its origins in childhood, with a low-grade systemic inflammatory condition arising from the large concentration of white adipose tissue^5^. The adipose tissue releases adipokines that contribute to reduced insulin sensitivity, reduced energy expenditure, increased leptin resistance, and several other molecular cascades with major long-term adverse health effects, including reducing the quality of life^8,10^ and contributing to premature mortality^5,8^. Improving the overall picture of obesity, not just weight loss, has been a major challenge for researchers and health professionals^19^. As such, several strategies have been presented in the literature to promote and sustain well-being over time^12,36,37^. Although exercise is a recommended strategy^8,16^, the novelty aspect of the present study was the manipulation of the variables of the intensity of combined training (i.e., training progression) to understand the impact of this on the HRQoL of this population.

The benefits of combined exercise on muscle structure, cardiorespiratory fitness, reducing systemic inflammation and metabolic markers^8,16,17^, as well as the reduction of articulate and back pain^23^ in people with obesity are already evident. The improvement in functional capacity in the groups that performed the combined training was due to the aforementioned improvements. However, the different training prescriptions did not provide different results in this domain. The benefits of increased intensity, observed in studies with aerobic or strength exercises performed in isolation^20,38^, may be linked to the different forms of manipulation of the training load, with greater amplitudes than those used in the present study (40–69% HRres; 14-8 RM). This may explain the similar improvements observed in some PCS domains, regardless of training progression. In addition, increased muscle strength has a greater impact on the quality of life of people who are frail in this physical capacity, such as the elderly^39,40^.

Studies that have examined the effect of combined training on HRQoL, including overweight people, have reported conflicting results. Sillanpää et al.^28^, observed an improvement in general health only among PCS, with a tendency to worsen the domains of role-physical and bodily pain in adults. In contrast, Goldfield et al.^26^, when analyzing the effect of training on adolescents, reported a significant improvement in functional capacity. In contrast, Baptista et al.^39^, when evaluating the HRQoL of elderly people, observed improvement in three of the four physical domains (except role-physical), in addition to the PCS itself. While Chang et al., observed only a trend of improvement in PCS after 3 months of intervention in elderly people with sarcopenic obesity^40^. It should be noted that the three of the four studies cited used some form of training progression, making it impossible to compare them with studies with fixed intensity methodologies. Furthermore, in the study by Chang et al., practitioners freely chose the intensity of exercise, so it is not possible to guarantee progression throughout the intervention period^40^. Therefore, the results of training interventions combined with and without progression are still inconclusive regarding their effects on HRQoL PCS.

The regular practice of exercise attenuates psychosocial disorders in different ways, either by improving the immune system and other physiological markers, or by the capacity for distraction and self-efficacy^22^. In addition, improvement results in the MCS may precede the physical benefits, as they need greater stimuli for their adaptation, while the feeling of belonging to the group, distraction from stressful environments, and a feeling of increased vigor provide psychological well-being, achieved even with reduced training volume^41–43^. Evidence has suggested that exercising 30 to 60 min, three to five times a week, reduces mental burden, improves aspects of mental health^42^, and in more severe disorders the practice is efficient, for example, improving depressive conditions^44^. It is worth mentioning that the studied population did not have any diagnosed psychological disorder. Even so, the training had positive effects on mental health. The improvement of this domain is of paramount importance in this population. Population-based evidence from the same region where the study was conducted pointed to a 45% increase in the prevalence of depressive disorders in the presence of CNCD^45^.

The low weekly adherence in training sessions and the consequent reduction in physical and mental components may be related to motivational aspects. Although the motivation is high at the beginning of a lifestyle intervention program, its maintenance is challenging^46^. During the process, the link between health and exercise is not well understood among adults with obesity, believing that health improvement is only linked to weight loss^47,48^. As this does not happen easily, individuals reduce their motivation to continue in the program, reducing their attendance rate and affecting their perception of health^47,48^. Additionally, the adherence observed corresponds to only one training session for some participants of the 3 days offered in the week. This would not be enough to induce the necessary physical adaptations for a noticeable improvement at the end of the intervention and could help to explain the decline in the physical component level of some participants^49^.

Recently, a cross-sectional study observed that physical activities with moderate intensities were more associated with high HRQoL compared to other levels of intensity^50^. This result is consistent with the improvement in the MCS found in the FG. However, the correlation presented in this study uses the level of significance to emphasize superiority, while the strength of correlation between the different intensities remains the same^50^, a result similar to that reported in the PCS between FG and LG. In a longitudinal study by Chekroud et al.^42^, higher intensities were associated with improved mental health in more than 1.2 million individuals. This corroborates the initial hypothesis that a program with linearly increasing intensity would present superior results in health parameters due to the increase in intensity^8,16,18,20,21^. Despite this, Reid et al.^27^, when verifying the impact of progressive combined training on patients with type II diabetes mellitus and excess weight, did not report an improvement in MCS, which, according to the authors, was due to the excessive fatigue of the modality. This corroborates the findings of the present study, since more expressive results were found only in the group with no progression of intensity for mental health and MCS and, even if not significant, in role-emotional, social functioning, and vitality. The difference in the frequency of training between the groups could explain the improvement of the MCS domains observed in the FG. Despite being 8.5% higher than the LG, there were no statistically significant differences, and thus, not a weighting factor for improvement^46^. Another consideration is the intrinsic factors linked to the practice of exercises, such as preferences for practice and motivation. These exercise have a direct influence on health parameters^46^, but they represent a limitation of the present study since they were not evaluated.

The applied single-blind randomized controlled trial is one of the main strengths of this study. Being an intervention lasting 16 weeks, using combined training with two forms of periodization, including a control group for comparison, with randomization and blinding are also positives. Likewise, the equalization of the training volume allows the different periods to be compared equivalently. The re-evaluation of the resting HR should be highlighted to adjust the participants’ internal load to maintain the proposed intensity. Another important factor was the study sample of individuals with obesity only, free of other comorbidities. This reduced the number of eligible candidates for the study but increased the representativeness of the HRQoL analysis in individuals with obesity.

This study has some limitations. The low participant adherence to the training sessions and the number of dropouts from the study must be considered when observing the results. The participants would likely benefit from greater effects by training more often^49^, with consequential implications for their HRQoL. Likewise, dropping out of the study may be due to low self-esteem, vitality, and other negative psychological factors present in this population^47^. Thus, more expressive results were not found in the PCS, possibly due to the low training attendance among the participants, especially given the need for frequent stimuli for the physiological adaptations to occur. Dropouts from this research may be less aware of their health status, which may have negatively affected their motivation^48^. It is important to recognize that satisfaction, preference, and pleasure when carrying out training are essential for the maintenance of individuals. Therefore, it is possible that the training proposal used, without adherence strategies, is not sufficient to motivate this population^46^. A systematic review demonstrated that interventions in people with obesity have dropouts similar to those found in this study^51^. Looking at the low MCS score, some individuals with depressive disorder, or at least with depressive symptoms, may have been included in the study. The search for professional help and the clinical diagnosis of individuals affected by mental conditions tend to occur over time. Since the perception of symptoms and the perception of worsening health are mediators in the process^52^, not recognizing the presence of these diseases when questioned initially is possible.

In conclusion, fixed-intensity combined training is effective in improving mental health domain and the mental health component. However, similar results were found in the physical component summary, regardless of training periodization. Future studies should investigate the relationship between the proposed periodization and the improvement of HRQoL, thereby contributing to a greater understanding of the benefits among those with obesity, including different forms of periodization, such as undulating periodization. Manipulations in the proposed training frequency, duration, and intensity may expand the knowledge about the possible HRQoL implications. Furthermore, future studies must consider the potentially low adherence to training in this population, and develop adoption strategies to maintain the participants’ adherence to exercise programs.

## Supplementary Information

Supplementary Table 1.

## Data Availability

The datasets used and/or analyzed during the current study are available from the corresponding author upon reasonable request.

## Abbreviations

HRQoL: Health-related quality of life
BMI: Body mass index
CG: Control group
FG: Fixed intensity group
LG: Linearly increasing group
HRres: Heart rate reserve
RM: Maximum repetitions
CNCD: Chronic non-communicable diseases
PCS: Physical component summary
MCS: Mental component summary
