# Supplementary material for: Effects of linear periodization of combined training on quality of life of adults with obesity: a blind randomized controlled trial
Source: Sci Rep. 2022 Feb 16;12:2567. doi: 10.1038/s41598-022-06461-8 (PMC8850548; doi:10.1038/s41598-022-06461-8)
Supplement: Supplementary file 1 — Supplementary Table 1. [file 41598_2022_6461_MOESM1_ESM.docx]

| **Supplementary table 1**. Comparison of the effects of training on Health-related quality of life with intention-to-treat (n=69). | | | | | | | | | | | | | | | |
| --- | --- | --- | --- | --- | --- | --- | --- | --- | --- | --- | --- | --- | --- | --- | --- |
|  | Control Group (n=23) | | | Non-Periodized Group (n=23) | | | Periodized Group (n=23) | | |  |  |  |  |  |  |
|  | Pre | Post |  | Pre | Post |  | Pre | Post |  | Group | | Time | | Group*Time | |
| **Components of SF-36** | $\bar{X}$ ± SE | | Δ | $\bar{X}$ ± SE | | Δ | $\bar{X}$ ± SE | | Δ | p valor | $\eta_{p}^{2}$ | p valor | $\eta_{p}^{2}$ | p valor | $\eta_{p}^{2}$ |
| **PCS** | 62,9 ± 3,5 | 59,7 ± 4,7 | -3,2 | 63,4 ± 2,8 | 64,6 ± 3,5 | 1,2 | 65,4 ± 3,6 | 71,2 ± 3,7 | 5,8 | 0,260 | 0,05 | 0,621 | 0,00 | 0,392 | 0,04 |
| Role-Physical | 68,5 ± 7,1 | 60,0 ± 9,1 | -8,5 | 70,5 ± 6,9 | 69,1 ± 9,6 | -1,4 | 68,5 ± 7,9 | 82,8 ± 7,6 | 14,3 | 0,369 | 0,02 | 0,802 | 0,00 | 0,306 | 0,07 |
| Physical functioning | 76,5 ± 3,8 | 75,7 ± 5,4 | -0,8 | 75,2 ± 4,0 | 81,8 ± 5,0 | 6,6 | 75,4 ± 3,9 | 82,5 ± 3,8 | 7,1 | 0,848 | 0,02 | 0,118 | 0,03 | 0,359 | 0,02 |
| Bodily pain | 63,5 ± 4,2 | 58,5 ± 5,4 | -5,0 | 61,5 ± 4,1 | 58,8 ± 5,1 | -2,7 | 66,8 ± 4,2 | 74,0 ± 5,0 | 7,2 | 0,073 | 0,09 | 0,963 | 0,00 | 0,303 | 0,03 |
| General health | 43,0 ± 4,1 | 44,7 ± 3,4 | 1,7 | 46,2 ± 3,0 | 48,5 ± 3,3 | 2,3 | 51,1 ± 3,5 | 45,3 ± 4,1 | -5,8 | 0,560 | 0,02 | 0,818 | 0,00 | 0,443 | 0,04 |
| **MCS** | 47,2 ± 4,7 | 57,6 ± 4,9 | 10,4 | 50,7 ± 4,1 | 65,8 ± 5,3 | 15,1 | 53,7 ± 4,7 | 62,0 ± 5,5 | 8,3 | 0,515 | 0,02 | <0,001 | 0,19 | 0,653 | 0,04 |
| Role-Emotional | 39,1 ± 7,8 | 62,2 ± 9,4 | 23,1 | 39,4 ± 8,5 | 70,6 ± 10,0 | 31,2 | 46,4 ± 8,6 | 68,8 ± 10,8 | 22,4 | 0,787 | 0,01 | <0,001 | 0,21 | 0,826 | 0,04 |
| Social Functioning | 54,9 ± 5,0 | 65,8 ± 5,7 | 10,9 | 64,8 ± 4,7 | 70,6 ± 6,9 | 5,8 | 62,5 ± 4,9 | 64,8 ± 6,4 | 18,4 | 0,494 | 0,02 | 0,134 | 0,05 | 0,689 | 0,02 |
| Mental health | 54,6 ± 4,8 | 59,2 ± 4,5 | 4,3 | 60,2 ± 4,0 | 68,9 ± 4,8 | 8,7 | 62,3 ± 4,3 | 61,3 ± 5,6 | -1,0 | 0,386 | 0,03 | 0,133 | 0,04 | 0,409 | 0,07 |
| Vitality | 40,2 ± 4,1 | 43,3 ± 4,8 | 3,1 | 38,6 ± 3,6 | 53,2 ± 4,3 | 14,6 | 43,7 ± 3,8 | 53,1 ± 5,8 | 9,7 | 0,475 | 0,04 | <0,001 | 0,17 | 0,160 | 0,02 |
| Note: Group= difference between groups; time= difference between times; Group*Time= interaction between time and group; $\bar{X}$= mean; SE = standard error; Δ= difference between post and pre-intervention; $\eta_{p}^{2}$= partial eta-squared; PCS = Physical Component Summary; MCS = Mental Component Summary. | | | | | | | | | | | | | | | |
